# Sensation of the tiniest kind: the antennal sensilla of the smallest free-living insect *Scydosella musawasensis* (Coleoptera: Ptiliidae)

**DOI:** 10.7717/peerj.10401

**Published:** 2020-11-23

**Authors:** Anna V. Diakova, Alexey A. Polilov

**Affiliations:** Department of Entomology, Faculty of Biology, Moscow State University, Moscow, Russian Federation

**Keywords:** Insects, Miniaturization, Ptiliidae, Sensilla, Antenna, Scydosella

## Abstract

Miniaturization is a major evolutionary trend prominent in insects, which has resulted in the existence of insects comparable in size to some unicellular protists. The adaptation of the complex antennal multisensory systems to extreme miniaturization is a fascinating problem, which remains almost unexplored. We studied the antennal sensilla of *Scydosella musawasensis* Hall, 1999 (Coleoptera: Ptiliidae), the smallest free-living insect, using scanning electron microscopy. The antenna of *S. musawasensis* bears 131 sensilla; no intraspecific variation in the number or position of the sensilla has been revealed. Nine different morphological types of sensilla are described according to their external morphological features and distribution: four types of sensilla trichodea, one type of sensilla chaetica, two types of sensilla styloconica, and two types of sensilla basiconica. Morphometric analysis of the sensilla of *S. musawasensis*, based on measurements of the lengths and diameters of sensilla and their location and number, showed the absence of significant differences between females and males. Comparative allometric analysis of *S. musawasensis* and larger Coleoptera showed that the number of sensilla and the size of sensilla chaetica decrease with decreasing body size. However, the number of the types of sensilla and the length and diameter of the multiporous sensilla basiconica revealed no correlation with the body size. Comparison of the acquired data with the results of our earlier study of the antennal sensilla of some of the smallest parasitic wasps is used to put forward hypotheses on the common principles of miniaturization of the antennal sensory systems of insects.

## Introduction

Antennae are present in a vast majority of Hexapoda (Schneider, 1964). The antennae are complex multimodal organs involved in such processes as the search for a sexual partner, a host, or food, or as intraspecific communication, enemy detection, orientation and navigation during flight, and many others (Altner, Sass & Altner, 1977; Guerenstein et al., 2004; Kamikouchi et al., 2009). The antennal sensory system consists of many components and has an extremely complex organization (Gao, Yuan & Chess, 2000; Xie et al., 2016). The number of antennal sensilla in insects can reach 100,000, and the number of their types can reach 17 (Shields & Hildebrand, 2001; Di Giulio et al., 2012). The study of the antennal sensilla of insects representive of various lineages and size classes started a long time ago, and the number and quality of such studies is growing with the advent and spread of new opportunities and techniques in microscopy (Callahan, 1975; Hallberg, Hansson & Lofsted, 2003). However, relatively few studies are devoted to the most speciose order of insects, Coleoptera. Most of those few studies use scanning electron microscopy and describe the external morphology of the antennal sensilla (Skilbeck & Anderson, 1996; Merivee et al., 2002). The results of such studies are often used for taxonomic purposes as diagnostic characters (Pérez-González & Zaballos, 2013). Even fewer studies analyze the internal structure and innervation of the sensilla (Moeck, 1967). Several studies deal with the involvement of the antennal sensory system in interspecies communication and behavioral responses in Coleoptera (Lopes, Marques & Araújo, 2005; Ali et al., 2016).

The evolutionary decrease in the body size, down to extreme miniaturization, is one of the principal directions of insect evolution and has recently been the subject of intense research (Polilov, 2015a; Minelli & Fusco, 2019). Body size largely determines the morphology, physiology, ecology, and behavior of insects (Eberhard & Wcislo, 2011; Polilov, 2016). Decrease in body size results in various morphological adaptations of the antennal sensory system. Thus, in Chalcidoidea, a correlation has been found between body size and the surface area of the antenna (Symonds & Elgar, 2013). A decrease in the number of antennomeres with a decrease in the body size has been shown in many of the smaller insects (Polilov, 2015a), down to only one antennomere in the male *Dicopomorpha echmepterygis* (Mockford, 1997) (Hymenoptera: Mymaridae). Allometric analysis has shown a decrease in the number of antennal sensilla and in the length of the mechanoreceptor sensilla with a decrease in the body length in parasitic wasps (Diakova, Makarova & Polilov, 2018). Studies on the intraspecific ranges of body sizes analyze the optimization of the antennal sensory system within the limits of intraspecific variation. A study on the olfactory system of the parasitic wasp *Trichogramma evanescens* Westwood, 1833 (Hymenoptera: Trichogrammatidae) revealed that the number and length of the olfactory antennal sensilla and the number of glomeruli in the antennal lobes positively correlates with the body size and that the absolute and relative sizes of the glomeruli correlate with the body length (Van der Woude & Smid, 2016). It has been shown that in the housefly *Musca domestica* Linnaeus, 1758 (Diptera: Muscidae), in which adult body size depends on the larval population density, the size of the antenna correlates with body size; moreover, although the antennae of larger and smaller flies bear the same types of sensilla, the antennae of larger houseflies bear nearly twice as many sensilla as those of smaller conspecifics (Smallegange, Kelling & Otter, 2008). A study on the size characteristics of worker ants of the species *Solenopsis invicta* Buren, 1972 (Hymenoptera: Formicidae) has shown that the length of the antennae relative to body length decreases with increasing body size (Tschinkel, Mikheyev & Storz, 2003).

The effects of extreme miniaturization on the antennal sensory system has barely been studied. We have analyzed earlier the antennal sensilla of three species of parasitic wasps of the genus *Megaphragma* Timberlake, 1924 (Hymenoptera: Trichogrammatidae), which have bodies only 0.2 mm long (Diakova, Makarova & Polilov, 2018). We found that due to miniaturization there is a significant decrease in the number of antennal sensilla: there are fewer than 50 sensilla per antenna in the studied species. Number and position of sensilla are the same in individuals of the same species and sex, which indicates a high degree of optimization of the antennal sensory system.

The effects of extreme miniaturization on the antennal sensory system in free-living insects have never been studied to date. The smallest free-living insect is *Scydosella musawasensis* (Hall, 1999) (Coleoptera: Ptiliidae), the body length of which is only about 0.3 mm (Polilov, 2015b). Only the general morphology of the antennae has been described in this species. The aim of this study is to analyze the arrangement and the structure of the antennal sensilla in *S. musawasensis.*

## Materials & Methods

### Materials

We used adult specimens of *Scydosella musawasensis* (Hall, 1999) (Coleoptera: Ptiliidae) collected for earlier projects (Polilov, 2015b). A total of 15 females and 12 males were fixed in the alcoholic Bouin liquid for studying the general morphology or in glutaraldehyde for studying ultrastructure.

### Scanning electron microscopy

The fixed material was gradually dehydrated through a series of ethyl alcohols and then acetone, critical point dried (Hitachi HCP-2), and sputtered with gold (Giko IB-3). The specimens were studied and photographed using Jeol JSM-6380 and FEI Inspect F50. The methods have been described in detail earlier (Diakova, Makarova & Polilov, 2018).

### Morphometry

All measurements were performed on SEM images, using the measurement tool in the Fiji package of ImageJ. Shapiro–Wilk normality test, descriptive statistics, ANOVA, SMA, and OLS were performed using R software (R Core Team, 2020). The *sma* and *slope.test* functions of the *smatr* package in R were used to determine the presence of allometry.

### Terminology

The nomenclature of the sensilla used in this study is described in Diakova et al. 2019; it is a system developed by us in accordance with earlier studies of the antennal sensilla of Staphylinoidea (Skilbeck & Anderson, 1996; Oliva, 2012).

## Results

### General description of the antenna of *S. musawasensis*

**Figure 1:**
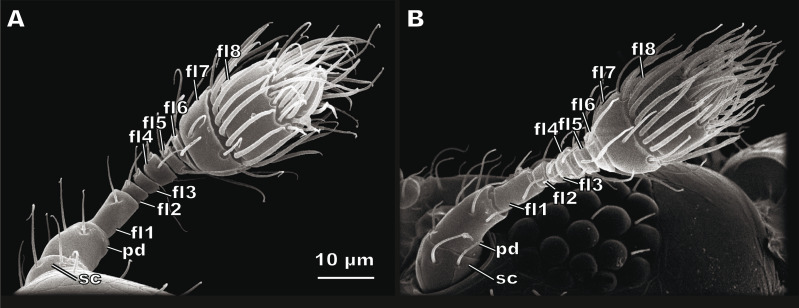
Female (A) and male (B) antennae of *Scydosella musawasensis*. sc, scape; pd, pedicel; fl1, flagellomere 1; fl2, flagellomere 2; fl3, flagellomere 3; fl4, flagellomere 4; fl5, flagellomere 5; fl6, flagellomere6; fl7, flagellomere 7; fl8, flagellomere 8.

**Figure 2:**
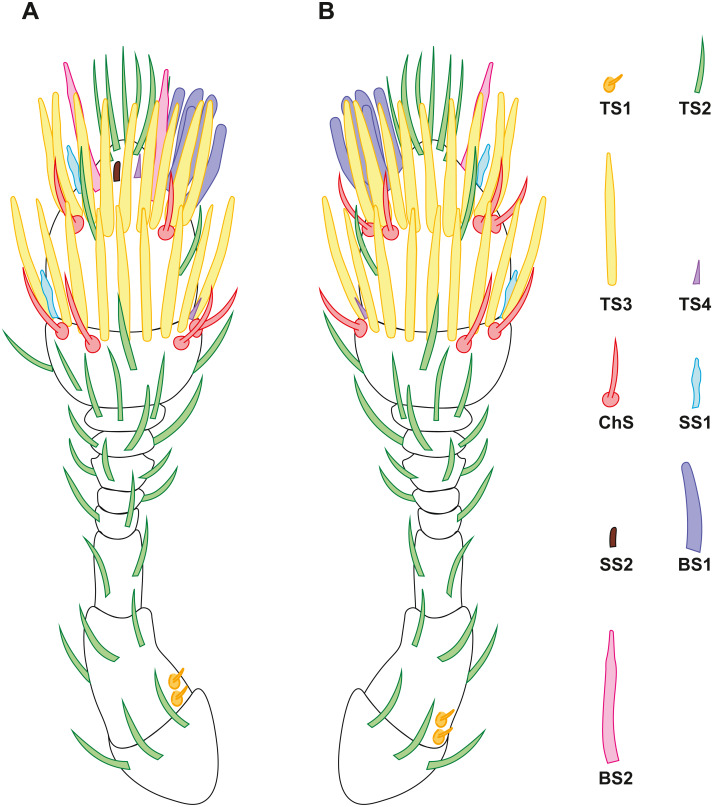
Distribution of antennal sensilla in *Scydosella musawasensis*. (A) Ventral view; (B) dorsal view.

**Figure 3:**
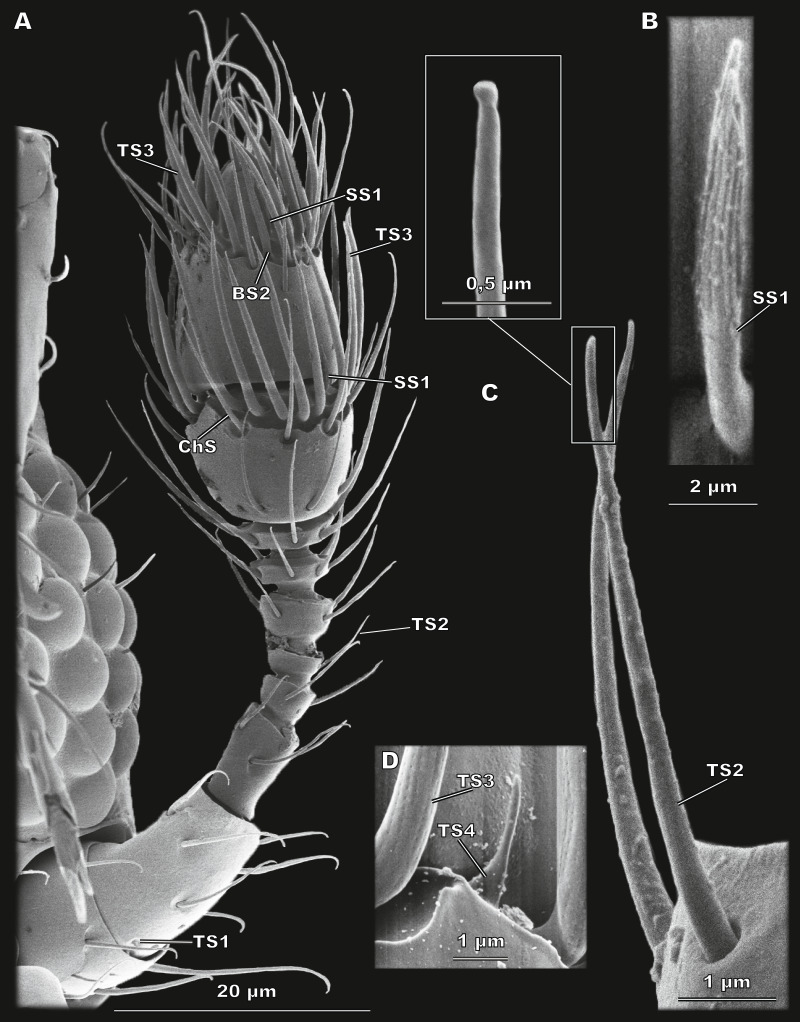
Morphological types of antennal sensilla in *Scydosella musawasensis* (SEM). (A) Whole antenna, ventral view, demonstrating TS1, TS2, TS3, ChS, SS1 and BS2; (B) aporous SS1 with longitudinal grooves; (C) TS2 with close-up showing its aporous tip; (D) aporous TS4 surrounded by TS3 with porous walls.

The antenna of *S. musawasensis* consists of ten antennomeres: the scape, the pedicel, and eight flagellomeres, which include the two-segmented club (Fig. 1). No differences were found in the structure of the antennae or shape of the antennomeres between the males and females. The antennae are 74.03 ± 2.93 µm long in the males and 73.02 ± 3.59 µm long in the females (Table 1). The antenna of *S. musawasensis* bears 131 sensilla (Figs. 2 and 3A). No intraspecific variation was found in the number and location of the sensilla.

**Table 1:** Length (L) and diameter (D) of antennal segments (mean ± sd) in *Scydosella musawasensis*, in µm.

	**sc L**	**sc D**	**pd L**	**pd D**	**fl L**	**fl D**	**club L**	**club D**
♂	9.21 ± 1.44	12.01 ± 0.93	14.32 ± 1.33	9.75 ± 0.67	50.49 ± 3.1	14.2 ± 1.27	27.7 ± 1.44	14.2 ± 1.27
♀	9.73 ± 1.23	12.21 ± 1.02	14.54 ± 1.45	8.75 ± 1.12	48.75 ± 2.81	15.26 ± 1.69	28.36 ± 2.02	15.25 ± 1.69

**Notes.**

sc scape pd pedicel fl flagellum

For additional details, see Table S1.

### The morphological types of the antennal sensilla and their distribution

Nine morphofunctional types of sensilla were identified based on the details of their external morphology, shape, and location. Since one-way ANOVA revealed no significant differences in the size of the sensilla between the males and females (Table S1), the mean values of the lengths and diameters of the sensilla of both sexes is given, ± standard deviation. No differences were revealed between the sexes also in the number of sensilla or in the number of their types.

#### Sensillum trichodeum type 1 (TS1)

**Figure 4:**
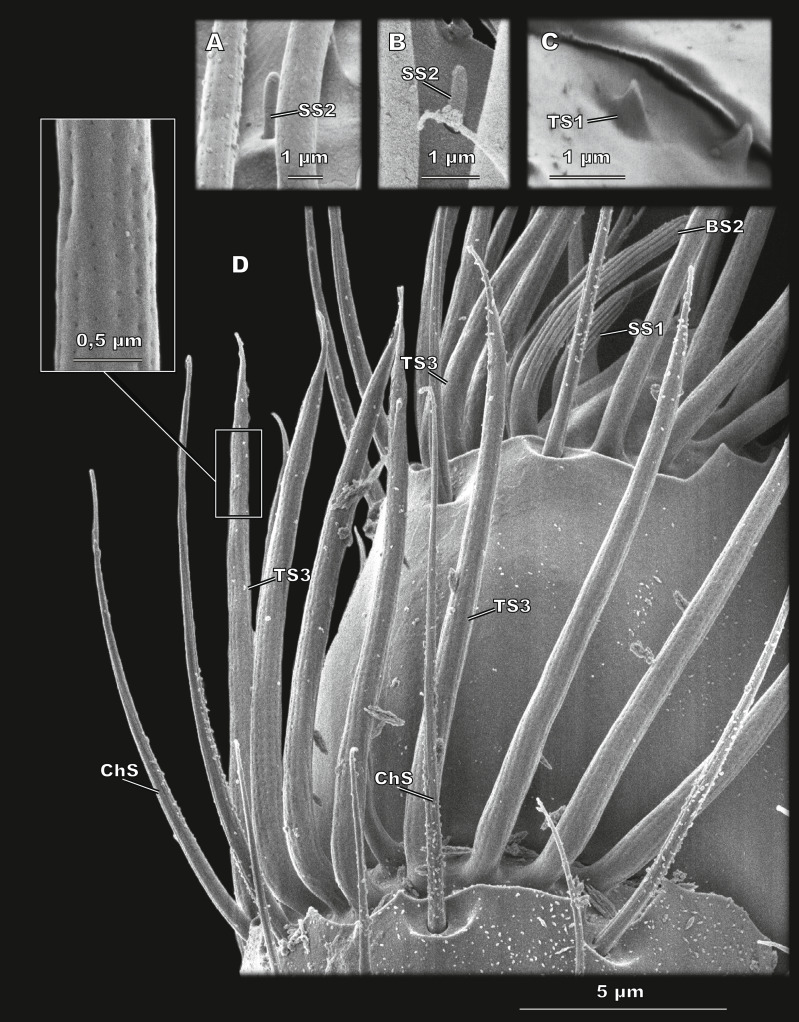
Club sensilla and proprioceptors of *Scydosella musawasensis* (SEM). (A) Aporous SS2 surrounded by porous TS3; (B) a small and inconspicuous SS2 is observed on the club; (C) Proprioceptive aporous TS1 situated on pedicel near the margin of scape; (D) detailed ventral view of the club. ChS, SS1, BS2, and TS3 with a close-up showing its porous wall are shown.

Very small aporous sensilla, each of which is located in a small depression (Fig. 4C). Four such sensilla are situated at the base of the pedicel (Fig. 2). This type of sensilla is the shortest, 0.63 ± 0.11 µm long. The diameter of TS1 is 0.44 ± 0.07 µm (Table 2; Table S1).

**Table 2:** Number (*n*) per antenna and length (L) and diameter (D) of antennal sensilla (mean ± sd) in *Scydosella musawasensis*, in µm.

	**Sensillum type**								
	TS1	TS2	TS3	TS4	ChS	SS1	BS1	BS2	SS2
***n***	4	61	38	2	14	2	1	7	2
**L**	0.63 ± 0.11	8.38 ± 1.98	13.14 ± 1.66	2.47 ± 0.7	9.61 ± 1.92	3.87 ± 0.72	7 ± 0.94	8.61 ± 1.1	0.72 ± 0.15
**D**	0.44 ± 0.07	0.52 ± 0.1	0.88 ± 0.11	0.44 ± 0.09	0.58 ± 0.06	0.6 ± 0.12	0.78 ± 0.08	0.71 ± 0.08	0.33 ± 0.05

**Notes.**

For more details, see Table S1.

#### Sensillum trichodeum type 2 (TS2)

Elongated aporous sensilla located in small cuticular depressions (Fig. 3C). The most numerous type of sensilla: each antennomere bears from two (flagellomere 3) to 15 (segment 2 of the club) TS2 (Fig. 2). There are a total of 61 TS2 on the antenna. TS2 are 8.38 ± 1.98 µm long and 0.52 ± 0.1 µm in diameter (Table 2; Table S1).

#### Sensillum trichodeum type 3 (TS3)

Large sensilla narrowing apically, with many pores arranged in rows over the entire surface (Fig. 4D). TS3 are located along the distal margin of segments 1 and 2 of the club, 20 and 18, respectively (Fig. 2). TS3 are the largest type of sensilla both in length (13.14 ± 1.66 *μ*m) and in diameter (0.88 ± 0.11 *μ*m) (Table 2; Table S1).

#### Sensillum trichodeum type 4 (TS4)

Small elongated poreless sensilla located at the distal margin of segments 1 and 2 of the club, one sensillum per segment (Figs. 3D and 2). Their length is 2.47 ± 0.7 µm, and their diameter is 0.44 ± 0.09 µm (Table 2; Table S1).

#### Sensillum chaeticum (ChS)

**Figure 5:**
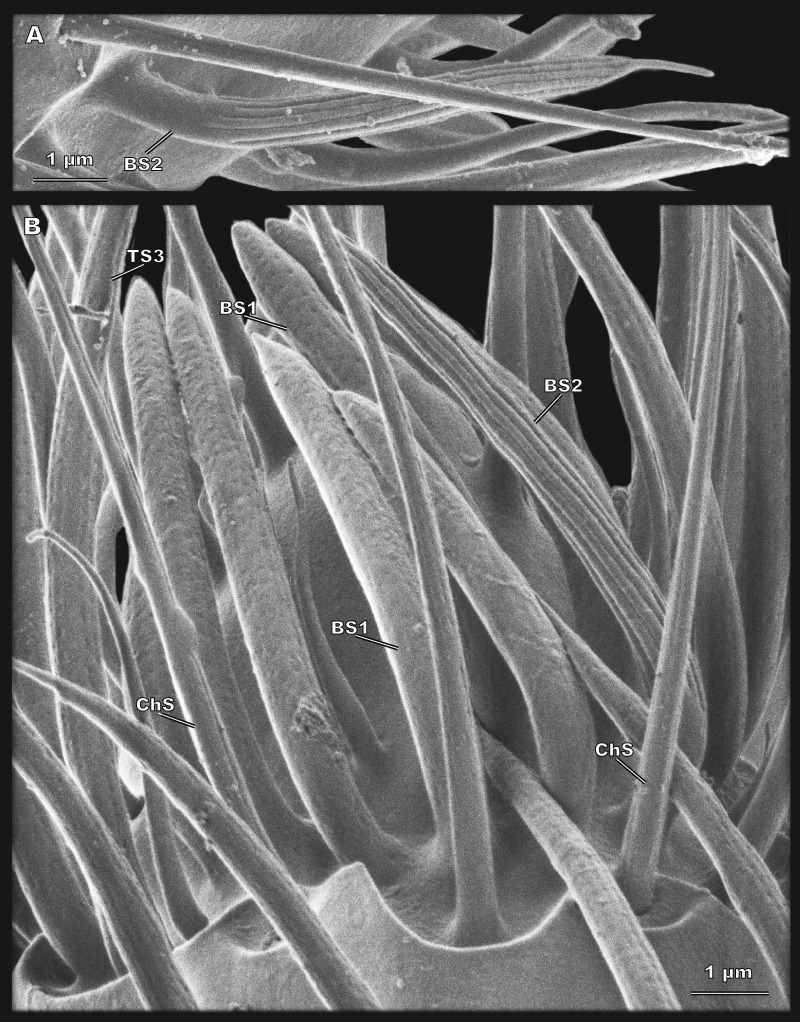
Sensilla of flagellomere 8 of *Scydosella musawasensis* (SEM). (A) BS2 with grooves and elongated tip situated on the club; (B) detailed medial view of segment 2 of the club. ChS with cuticular sockets, porous BS1 and TS3 and grooved BS2 are shown.

Elongated aporous sensilla with small longitudinal depressions, located in a wide and distinct cuticular socket (Figs. 4D and 5B). Arranged in a circle on segments 1 and 2 of the club, eight and six, respectively (Fig. 2). ChS are 9.61 ± 1.92 µm long and 0.58 ± 0.06 µm in diameter (Table 2; Table S1).

#### Sensillum styloconicum type 1 (SS1)

Small apically narrowing aporous sensilla with many longitudinal depressions in the apical half (Fig. 3B). One SS1 is located laterally on the distal edge of each of segments 1 and 2 of the club (Fig. 2). SS1 are 3.87 ± 0.72 µm long and 0.60 ± 0.12 µm in diameter (Table 2; Table S1).

#### Sensillum styloconicum type 2 (SS2)

Very small and inconspicuous aporous sensillum with a rounded apex (Figs. 4A and 4B). A single sensillum of this type is situated ventrally on segment 2 of the club (Fig. 2). SS2 have the smallest diameter, 0.33 ± 0.05 µm. Their length is 0.72 ± 0.15 µm (Table 2; Table S1).

#### Sensillum basiconicum type 1 (BS1)

Curved sensilla with a rounded tip and many pores all over the surface (Fig. 5B). Located in a group of seven sensilla on the medial surface of the segment 2 of the club (Fig. 2). BS1 are 7 ± 0.94 µm long and 0.78 ± 0.08 µm in diameter (Table 2; Table S1).

#### Sensillum basiconicum type 2 (BS2)

Large aporous sensilla with a narrow elongated apical narrowing and rows of pronounced longitudinal grooves (Figs. 5A and 5B). Two BS2 are located on segment 2 of the club, on the medial and lateral surfaces (Fig. 2). Their length is 8.61 ± 1.1 µm, and their diameter is 0.71 ± 0.08 µm (Table 2; Table S1).

## Discussion

### The structure of the antenna

The structure of the antenna of *S. musawasensis* is typical of the tribe Nanosellini. Most species of the family Ptiliidae have 11 antennomeres, which is the most common number of antennomeres in Staphylinoidea, but in *S. musawasensis,* and in many other miniature representatives of Nanosellini, the number of antennomeres is reduced to ten (Sörensson, 1997; Hall, 1999; Polilov et al., 2019).

### The morphological types of the antennal sensilla and their supposed functions

The antennae of Coleoptera usually bear many different types of sensilla; the greatest number of types of antennal sensilla among insects was described in a representative of this order, *Rhynchophorus palmarum* Linnaeus, 1758 (Coleoptera: Curculionidae) (Di Giulio et al., 2012). The antennal sensilla of Coleoptera are represented by many morphological types, in some cases unique to the species described (Newman, McDonald & Triebold, 1993). In the case of *S. musawasensis*, most of the revealed types of sensilla have been previously described in Coleoptera and have a typical structure and location. The few exceptions are TS4, SS2, and BS2.

#### Sensillum trichodeum type 1

Sensilla of this type, also known as the “Böhm bristles” (Böhm, 1911), are proprioceptors found in many insects, including the previously studied species of the genus *Aleochara* Gravenhorst, 1802 (Coleoptera: Staphylinidae), in which they have been found not only on the scape and pedicel, but also on the flagellum (Skilbeck & Anderson, 1996). They are usually located in groups on the articular surfaces between antennomeres. The movement of one of the antennomeres deflects the cuticular hair, signaling the position of the antennomeres relative to each other (Schmidt & Smith, 1986).

#### Sensillum trichodeum type 2

This is the most abundant type of sensilla on the antenna of *S. musawasensis.* A small depression at the base of the sensilla suggests a mechanosensory function (Zhantiev, 1969). The ultrastructural organization of such sensilla usually includes the dendrite of the sensory neuron, which reaches the cuticular depression and thus perceives the deviations of the long cuticular hair when it touches external objects (Consoli, Kitajima & Parra, 1999). These are common sensilla, found, among other insects, in many coleopterans (Payne et al., 1973; Merivee et al., 2002; Di Giulio et al., 2012).

#### Sensillum trichodeum type 3

The many regular pores on the cuticular surface of these sensilla indicate their olfactory function (Ivanov, 2000). Many branched dendrites of sensory neurons are usually located inside such sensilla. Similar sensilla have been described in the parasitic wasps *Tetrastichus hagenowii* Ratzeburg, 1852 (Hymenoptera: Eulophidae) and *Trichogramma brassicae* Bezdenko, 1968 (Hymenoptera: Trichogrammatidae), in which they are also located distally on the antenna (Barlin, Vinson & Piper, 1981; Romani, Isidoro & Bin, 2010). Sensilla similar in morphology and location have also been found in 16 species of Scolytidae (Coleoptera), in which they were described as sensilla basiconica long type (Payne et al., 1973).

#### Sensillum trichodeum type 4

These small inconspicuous sensilla resemble TS1 in structure, but they have no cuticular depression at the base and are located on the club, rather than on the articular surfaces. These characteristics suggest that these sensilla are not proprioceptors. There are no published data on this type of sensilla, but due to their small size it is possible that they were overlooked in larger insects. Their ultrastructure has to be studied to accurately determine their function.

#### Sensillum chaeticum

The sensilla chaetica of *S. musawasensis* appear as typical insect mechanoreceptors: they have a pronounced cuticular socket at the base and a long bristly cuticular hair and are located at an obtuse angle to the antennal axis (Ivanov, 2000). Such sensilla are known in most insects, including even the extremely simplified miniature male of *D. echmepterygis*, in which the only sensillum on the miniaturized antenna is a sensillum chaeticum (Mockford, 1997). Sensilla chaetica have been found in many species of Coleoptera (Dyer & Seabrook, 1975; Harbach & Larsen, 1977; Handique et al., 2017).

#### Sensillum styloconicum type 1

Such sensilla are found in many very different insects, usually located on the club, if it is present, or distally on the antenna, as in *S. musawasensis* (Altner & Loftus, 1985; Chiappini, Solinas & Solinas, 2001; Diakova, Makarova & Polilov, 2018). Sensilla of this type usually contain processes of several neurons and are thermo/hygroreceptors (Yokohari, 1999). Such sensilla have been described in some coleopterans; usually only one such sensillum is present, or very few of them (Smith, Carolina & Frazier, 1976).

#### Sensillum styloconicum type 2

Such sensilla are barely mentioned in the available publications. A similar sensillum has been found in *Oxelytrum erythrurum* Blanchard, 1849 (Coleoptera: Silphidae), in which it was also located on the ventral surface of the club (Oliva, 2012). The lack of data on their ultrastructure makes it impossible to propose a possible function for them at the moment.

#### Sensillum basiconicum type 1

The multiporous sensilla basiconica are a typical example of olfactory receptors found in many insects, including coleopterans (Borden, 1968; Kim & Yamasaki, 1996). In a study on the honeybee *Apis mellifera* Linnaeus, 1758 (Hymenoptera: Apidae), it was found that they are distance chemoreceptors (Lacher, 1964). They contain multiply branching processes of many sensory neurons (Chiappini, Solinas & Solinas, 2001).

#### Sensillum basiconicum type 2

These sensilla are very similar in shape and location to BS1, but the absence of pores on their surface suggests that they are not chemoreceptors. They could be thermo/hygroreceptors, but their exact function cannot be determined without studying their ultrastructure.

### The effects of miniaturization on the antennal sensilla of Coleoptera

Analysis of the dependence of the principal morphometric characteristics of coleopteran antennae on body size revealed the consequences of reduction in the latter and especially of extreme miniaturization. For the allometric analyses, in addition to the data obtained on the sensilla of *S. musawasensis*, we used published data from 16 sources on 33 species of beetles of eight families (Table S2); the body sizes of the beetles included in our analyses ranged from 0.3 to 40.0 mm (for references, see Appendix 1). We used the equation *y* = *ax*^*b*^, where *y* is the variable in question, a is elevation, *x* is body length, and *b* is slope (terminology follows the one described in Warton et al., 2006).

**Figure 6:**
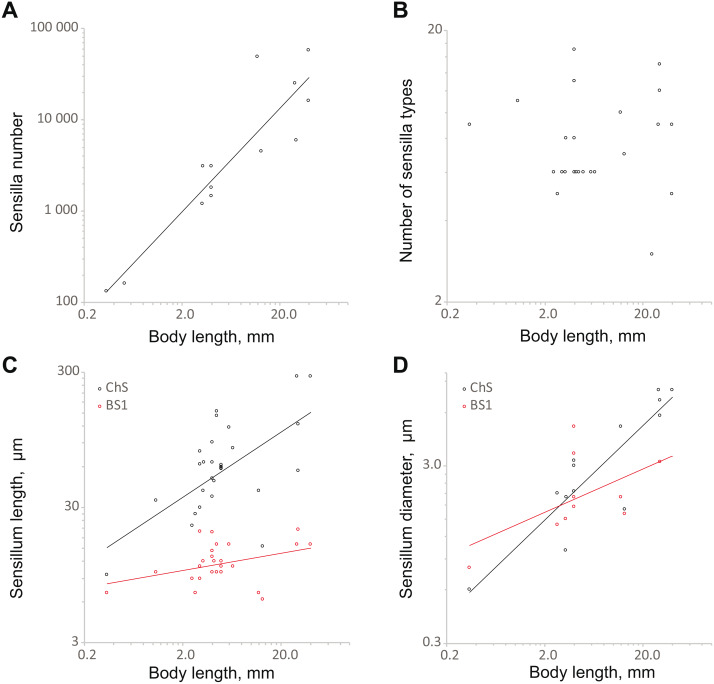
Changes in antennal sensilla properties in Coleoptera. (A) Number of antennal sensilla as a function of body length; (B) the number of antennal sensilla types as a function of body length; (C) mechanoreceptive ChS and olphactory BS1 length as a function of body length; (D) ChS and BS1 diameter as a function of body length. For references, see Table S3.

The number of sensilla correlates positively with body size (SMA 358.1*x*^1.31^, OLS 473.1*x*^1.16^; the slopes of both regressions are significantly different from 0, *p* < 0.01) (Fig. 6A; Table S3). The model explains a majority of the variability the data (*R*^2^ = 0.79 for SMA and OLS). The range is from 131 (*S. musawasensis*) to 57 370 sensilla (*Rhynchophorus palmarum* Linnaeus, 1758, Coleoptera: Curculionidae) per antenna (Saïd et al., 2003). Decreasing numbers of antennal sensilla with decreasing body size have been shown earlier in parasitic wasps (Diakova, Makarova & Polilov, 2018). Comparisons of larger and smaller individuals of *T. evanescens* (Van der Woude & Smid, 2016) and *M. domestica* (Smallegange, Kelling & Otter, 2008) also revealed a correlation between the body size and the number of antennal sensilla. The number of sensilla types in the analysis showed no significant correlation with body size (Fig. 6B; Table S3), same as found in parasitic wasps (Diakova, Makarova & Polilov, 2018). In a study on the intraspecific variation of *M. domestica,* no variation in the number of the types of sensilla was found either (Smallegange, Kelling & Otter, 2008). The base length and diameter of ChS show positive correlations with body size (length: SMA 14.55*x*^0.81^, OLS 27.99*x*^0.42^; diameter: SMA 0.91*x*^0.60^, OLS 1.04*x*^0.54^) (Figs. 6C and 6D; Table S3). The slopes for both length and diameter are significantly smaller than 1, *p* < 0.01, which indicates allometry and an increase in the relative sizes of ChS with decrease in body size. The model explains a majority of the variation in ChS diameter (*R*^2^ = 0.79). However, model for ChS length demonstrates low *R*^2^ = 0.27, which implies a possibility that the model is imprecise. The ranges are 9.6 to 280.0 µm for length and 0.6 µm to 8.0 µm for diameter. Length and diameter of BS1 showed no significant correlation with body size (Figs. 6C and 6D; Table S3); the slope did not significantly differ from 0, which indicates that the sizes of sensilla do not change with decreasing body size. In the study on the antennal sensilla of parasitic wasps, a weak correlation of the length of ChS with the body length has been revealed, but no correlation has been found between the diameter of ChS and body length or between the size characteristics of BS1 and body length (Diakova, Makarova & Polilov, 2018).

### Comparison of the effects of extreme miniaturization on the antennal sensilla in the free-living *S. musawasensis* vs. the parasitic wasps of the genus *Megaphragma*

Comparison with the results of our earlier study on the antennal sensilla of three species of miniature parasitic wasps of the genus *Megaphragma* (Diakova, Makarova & Polilov, 2018) reveals universal adaptations of the antennal sensory systems to extreme miniaturization common to parasitic and free-living insects. As in *S. musawasensis*, very few antennal sensilla were found in *Megaphragma* wasps. Species of the genus *Megaphragma* have 39–49 antennal sensilla per antenna (depending on the species and sex), while each antenna of larger parasitic wasps can bear up to 9000 sensilla (Das et al., 2011). In both beetles and parasitic wasps the number of antennal sensilla decreases with decreasing body size.

No correlation between the body size and the number of the types of sensilla has been revealed in either beetles or parasitic wasps. The diversity of sensory information perceived by the insects depends on the number of the types of antennal sensilla. A small number of types and subtypes of sensilla indicates a low diversity of sensory information, which usually includes tactile information and detection of temperature and humidity (Kapoor, 1985; Gaino & Rebora, 1998; Derr & Cook, 2005; Fialho et al., 2014). Insects with many types of sensilla, regardless of body size, have the entire possible spectrum of receptors: mechanoreceptors, contact and distant chemoreceptors, thermo/hygroreceptors, and proprioceptors.

The scaling of the dimensional characteristics of the sensilla depends on their type: while for ChS in both beetles and parasitic wasps a correlation of the length and diameter with the body size has been shown, for BS1 no significant correlation has been found. The reason why ChS are scaled but BS1 are not could be the greater complexity of the internal structure of BS1. Neurons containing specific olfactory receptor proteins, their projections, and the glomeruli of the antennal lobe constitute a complex system with an odotopic organization (Gao, Yuan & Chess, 2000), which is difficult to optimize and miniaturize. It is probably the complexity of the internal structure that limits the optimization of the olfactory BS1, as a result of which there is no correlation between their size characteristics and the body size.

## Conclusions

A decrease in body size leads to a significant decrease in the number of individual sensory units (sensilla) in the antennal sensory systems of both parasitic and free-living insects. A similar trend is also found in the miniaturization of the compound eyes of insects, for which the number of ommatidia has been shown to decrease significantly with decreasing body size. At the same time, miniature ommatidia show a cellular organization similar to that of larger insects and a number of structural adaptations associated with miniaturization (Makarova, Meyer-Rochow & Polilov, 2019). The scalability of sensilla during miniaturization depends on the type of the sensilla. Mechanosensory sensilla are more scalable, while olfactory sensilla, which have a more complex ultrastructure, do not get smaller with decreasing body size. The number of functional types does not correlate with body size, which indicates the preservation of the complexity and functionality of the antennal sensory system in most of the smallest insects studied. Thus, a miniature antenna is a highly functional multimodal organ with a small number of different receptors, which respond to a wide range of different sensory information.

##  Supplemental Information

10.7717/peerj.10401/supp-1 Table S1 Raw measurements of Scydosella musawasensis antennal sensilla

10.7717/peerj.10401/supp-2 Table S2 References for allometric analysis

10.7717/peerj.10401/supp-3 Table S3 Analysis of the dependence of the morphometric characteristics of coleopteran antennae on body size We used the equation *y* = *ax*^*b*^, where *y* is the value in question, a is elevation, *x* is body length, and *b* is slope. Slope, *R*^2^ and elevation values were calculated for two regression types, SMA and OLS. ChS, Sensillum chaeticum; BS1, Sensillum basiconicum type 1. *–variables correlate and *b* is significantly different from 0 (*p* < 0.01).

## Additional Information and Declarations

### Data Availability

The following information was supplied regarding data availability: The raw measurements are available in the Supplementary File.
